# Kinetochore tracking in 3D from lattice light-sheet imaging data with KiT

**DOI:** 10.1093/bioinformatics/btac330

**Published:** 2022-05-17

**Authors:** Jonathan U Harrison, Onur Sen, Andrew D McAinsh, Nigel J Burroughs

**Affiliations:** Zeeman Institute (SBIDER), Mathematics Institute, University of Warwick, Coventry, UK; Centre for Mechanochemical Cell Biology and Division of Biomedical Sciences, Warwick Medical School, University of Warwick, Coventry, UK; Centre for Mechanochemical Cell Biology and Division of Biomedical Sciences, Warwick Medical School, University of Warwick, Coventry, UK; Zeeman Institute (SBIDER), Mathematics Institute, University of Warwick, Coventry, UK

## Abstract

**Motivation:**

Lattice light-sheet microscopy (LLSM) is revolutionizing cell biology since it enables fast, high-resolution extended imaging in three dimensions combined with a drastic reduction in photo-toxicity and bleaching. However, analysis of such datasets still remains a major challenge.

**Results:**

Automated tracking of kinetochores, the protein complex facilitating and controlling microtubule attachment of the chromosomes within the mitotic spindle, provides quantitative assessment of chromosome dynamics in mitosis. Here, we extend existing open-source kinetochore tracking software (KiT) to track (and pair) kinetochores throughout prometaphase to anaphase in LLSM data. One of the key improvements is a regularization term in the objective function to enforce biological information about the number of kinetochores in a human mitotic cell, as well as improved diagnostic tools. This software provides quantitative insights into how kinetochores robustly ensure congression and segregation of chromosomes during mitosis.

**Availability and implementation:**

KiT is free, open-source software implemented in MATLAB and can be downloaded as a package from https://github.com/cmcb-warwick/KiT. The source repository is available at https://bitbucket.org/jarmond/kit (tag v2.4.0) and under continuing development.

**Supplementary information:**

Supplementary data are available at *Bioinformatics* online.

## 1 Introduction

Human somatic cells divide by mitosis, whereby each sister in 46 pairs of chromosomes must become attached to opposite poles of the mitotic spindle, congress to the cell equator and segregate towards the spindle poles (Maiato *et al.*, 2017; Vukušić *et al.*, 2019). In order to gain full quantitative understanding of the processes governing dynamics of chromosomes throughout mitosis, automated tracking is necessary. Improvements in light microscopy mean that live imaging of human cells in three dimensions over time and over multiple channels is now widespread. However, to make best use of these data requires automated image analysis tools to perform tracking of kinetochores, the protein complex located at the centromere that facilitates and controls microtubule attachments between chromosomes and the mitotic spindle.

We demonstrate near-complete tracking of the 46 chromosome pairs in a cell, made possible by improvements to KiT tracking software (Armond *et al.*, 2016) described here, and by the high signal-to-noise ratio, and lower bleaching rate achievable with lattice light-sheet microscopy (LLSM). We provide assessment of the performance of the tracking algorithm over long timescales across the metaphase–anaphase transition at multiple temporal resolutions.

Another key challenge lies in ensuring that tracking software is robust to variation in input images across different imaging conditions and to diagnose which part of a tracking pipeline is limiting analysis of a specific dataset. To address this, we have developed a suite of detailed diagnostics to help a user identify and address the factors that limit tracking coverage, and assess the performance of tracking.

## 2 Materials and methods

Most tracking algorithms consist of two key steps: (i) detection of objects within a frame and (ii) linkage of objects between frames (Jaqaman *et al.*, 2008). In the context of kinetochore dynamics, additional steps are also required to (iii) define a relevant biological coordinate system and (iv) to group together kinetochore sister pairs, which are physically connected by a centromeric chromatin spring up to anaphase, but are detected and tracked as two separate objects.

Here, detection proceeds by identifying candidate spots from local intensity maxima and setting a threshold on the histogram of image intensities to separate signal from background. The threshold is identified via either (i) unimodal histogram thresholding (Rosin, 2001), or (ii) an adaptive method based on similarity between point clouds (Armond *et al.*, 2016). Locations of candidate spots detected via this initial step are refined by either (iii) fitting a mixture of 3D Gaussians to the spot locations (Thomann *et al.*, 2002) (which helps disambiguate overlapping spot locations and can provide sub-pixel resolution of spot locations), or (iv) taking the centroid of a region of interest around a spot location (Jaqaman *et al.*, 2010).

A plane is fitted to provide a relevant biological coordinate system, based on eigenvectors of the covariance matrix of 3D spot locations. This corresponds to the metaphase plate, which lies at the cell equator perpendicular to the spindle axis.

Linkage of spots between frames relies upon solving a linear assignment problem (Jaqaman *et al.*, 2008) between spot locations in successive frames, with motion propagation via a Kalman filter. Tracks of individual kinetochores are grouped together to form kinetochore pairs by solving another linear assignment problem based on summary statistics including the average distance between kinetochores, variance of the distance between kinetochores and alignment of the inter-kinetochore vector with the normal to the metaphase plate.

## 3 Results

We describe two key improvements compared to previous versions of this tracking algorithm (Armond *et al.*, 2016; Jaqaman *et al.*, 2008, 2010; Vladimirou *et al.*, 2013). First, we introduce a regularization term in the objective function for the adaptive detection step whereby we include biological knowledge about the number of kinetochores as follows:

$$J=\frac{1}{T-1}\sum_{t=1}^{T-1}m(s_{t},s_{t-1})-\lambda \frac{1}{T}\sum_{t=1}^{T}|n(s_{t})-n_{e}|,$$

where *s_t_* are the proposed locations of the $n(s_{t})$ spots in frame *t* of the movie, $m(s_{t},s_{t-1})$ is the mean minimum difference between spot locations and *n_e_* is the biologically expected number of kinetochore spots (92 in human cells) such that $\left|n(s_{t})-n_{e}\right|$ measures the discrepancy from the expected number of spots in frame *t*, with regularization parameter, *λ*. The objective function, *J*, is then minimized over choices for the threshold on the image histogram, which dictates the number and locations of spots, *s_t_* in each frame. This objective function ensures that close to the expected number of spots are detected in each frame. Adding this biologically informed constraint ensures robustness of the detection and tracking methods; when using alternative regularization terms, movies with low signal-to-noise ratios can result in detection of thousands of candidate spots, which renders the Gaussian mixture model computationally infeasible.

**Fig. 1.**
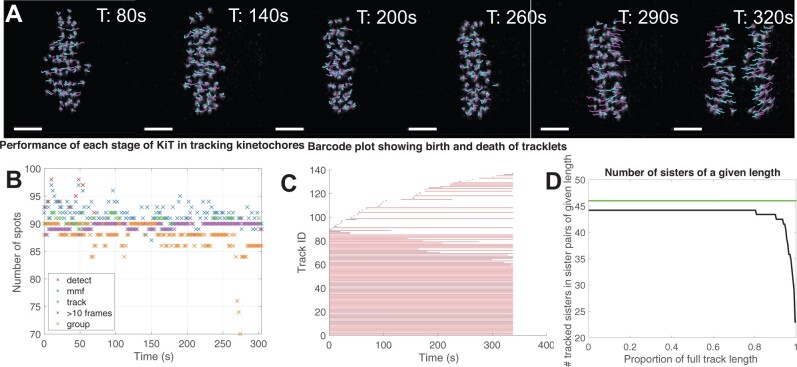
Near-complete tracking of kinetochores through metaphase–anaphase transition in human RPE1 cells. (**A**) Sequence of images [as in Sen *et al.* (2021)] showing metaphase–anaphase transition with dragontails indicating tracked and paired kinetochores. Magenta (cyan) lines show trajectories over the next (previous) five frames. (**B**) The number of spots detected and tracked at each stage of the algorithm quantifying performance at each step of the algorithm and over the duration of the movie. (**C**) Barcode plot showing birth and death of kinetochore tracks. (**D**) The number of kinetochore pairs tracked through at least a given proportion of the duration of a movie. Green line indicates 46 kinetochore pairs (A color version of this figure appears in the online version of this article.)

Second, we provide an option to use global background subtraction rather than local background subtraction. Estimating background locally, such as determined by convolving with a large 3D Gaussian kernel (Jaqaman *et al.*, 2010), can be helpful in settings with structure in the background signal, for instance generated by cytoplasm auto-fluorescence. However, for LLSM data with uniform background such local background estimation can itself create structure in the background when none is present, which reduces spot detection accuracy. Together these two improvements to the tracking algorithm result in a >2-fold increase in the number of kinetochore pairs tracked for >75% of a movie across a dataset of five movies, representing varied imaging conditions, from a total of 67 pairs to 152 pairs. Near-complete tracking of all kinetochore pairs in a cell is thus possible, e.g. with 44 out of 46 pairs tracked for >75% of the movie (2.05 s per frame, 170 frames) for the cell in Figure 1A.

Diagnostic tools enable users to identify in what ways the tracking software may be performing sub-optimally, and what specific changes can be made to imaging protocols to address this. Some of the diagnostics are shown in Figure 1, including how many kinetochores are detected/lost at each stage of the algorithm (Fig. 1B), the number of kinetochores tracked through a given portion of the movie (Fig. 1C) and barcode plots (Fig. 1D) showing complete/partial tracks and where these break down. Interpretation of these plots can offer insight into whether spot detection or linkage of spots between frames is the limiting step, reveal effects of biological events (such as anaphase onset) on tracking, or other changes in spot intensity over time such as bleaching or protein dynamics. For example, numbers of detected kinetochores close to *n_e_* but numbers of tracked kinetochores close to 0 likely indicates a problem with linking spots between frames. Such a problem could be a result of incorrectly entering the time resolution, Δt, or could mean that the data itself has too low temporal resolution. Further troubleshooting suggestions based on these diagnostic plots are provided in Supplementary Material and in the docs folder of the software on GitHub.

## 4 Usage

A graphical user interface is provided for ease of use, activated by typing the command kitrun; further details on usage are given in the manual provided with the software. Installation is not required, and the bioformats library (Linkert *et al.*, 2010), which can be automatically downloaded if required, is used to load multiple formats of image data.

## 5 Discussion

In this work, we present software for near-complete tracking of kinetochores in mitosis. Through changes to the spot detection step, we ensure robustness to a broad range of imaging conditions. Finally, by improving the diagnostic tools available to users, we enable better understanding of how tracking a movie can go wrong, leading to iterative improvement of tracking for users. Future development of this software can also be guided by understanding its limitations.

The main ways in which the tracking algorithm can fail are (i) no or very few tracks found in a cell (in which case the step where loss occurs can be diagnosed with the diagnostic plots and thus addressed by altering algorithm parameters or imaging conditions as appropriate), or (ii) linking incorrect kinetochores between frames resulting in label switching, which can be identified by visualizing dragontail plots as in Figure 1A. Incorrect detections of (random) background noise will not be linked across multiple frames and thus will not form long tracks. In this way, linking a track together provides a self-check built into the algorithm.

Sufficiently high time resolution is essential for high quality tracking. Given that human kinetochores move with speeds up to 50 nm/s, we found that data imaged at a temporal resolution of 2–4 s gave reliable results. With a larger time step (8 s or more) between frames, kinetochores move much further between frames and there is more uncertainty about which kinetochore has moved to which (detected) new location. The linkage step of the tracking algorithm may then (incorrectly) link different kinetochores between frames resulting in a label switch from one kinetochore to another. We therefore recommend using data with a temporal resolution of around 4 s per frame or less with this software, as this error then occurs with low frequency. In different species, adjustments may be needed for kinetochore speed and density. Brightness of spots relative to background is another key requirement to ensure that spots can be detected reliably; if a spot cannot be detected in a frame, then there will be no spot to link to for that track. Presence of prometaphase/metaphase movie frames are also needed to establish which kinetochores are sister pairs (although tracking of individual kinetochores can be performed without prometaphase/metaphase frames).

A key part of the improvement on LLSM data comes from avoiding detection of a high number of non-biological spots, which are often associated with the boundary of the image due to deskewing the image stack [transforming to traditional *x*, *y*, *z* image coordinates from the rotated frame for LLSM imaging (Chen *et al.*, 2014)]. This does not apply for data from other microscopy approaches. For movies from traditional (e.g. spinning disk confocal) microscopy where the number of spots detected differs from *n_e_* by an order of magnitude, we would expect to see a notable improvement from the new regularization term in the objective function, but otherwise may find little difference. Thus, this robust regularization term described here can be used in conjunction with other imaging approaches, but the benefits of doing so are much smaller than in the LLSM case.

Ultimately, this software aims to allow complete analysis of the positions of all 46 chromosomes simultaneously throughout mitosis in human cells. Near-complete tracking of kinetochores will allow the analysis of chromosomes dynamics, how these dynamics are controlled, and how errors are corrected throughout mitosis, from congression to chromosome segregation. Tracking the complement of kinetochores will ensure that conclusions are fully representative of mitotic dynamics and allow accurate determination of (rare) event rates and dynamics. This thus avoids bias through studying the behaviour of only a subset of chromosomes.

## Funding

This work was supported by the Biotechnology and Biological Sciences Research Council (BBSRC) [BB/R009503/1 to J.U.H., O.S., N.J.B. and A.D.M.]; and a Wellcome Senior Investigator Award [106151/Z/14/Z to A.D.M.]. The LLSM Facility was established at Warwick with a Wellcome Trust Multi-user Equipment grant to A.D.M. [208384/Z/17/Z].

*Conflict of Interest*: none declared.

## Data availability

Software available from https://github.com/cmcbwarwick/KiT. Data available on request.

## Supplementary Material

btac330_Supplementary_Data
