# Supplementary material for: Kinetochore tracking in 3D from lattice light-sheet imaging data with KiT
Source: Bioinformatics. 2022 May 17;38(12):3315–7. doi: 10.1093/bioinformatics/btac330 (PMC9191203; doi:10.1093/bioinformatics/btac330)
Supplement: btac330_Supplementary_Data [file btac330_supplementary_data.pdf]

TROUBLESHOOTING.md

KiT troubleshooting and diagnostics

This document details troubleshooting methods and diagnostic plots to help diagnose issues with tracking kinetochores, and suggest ways to improve tracking.
These will be demonstrated using a test dataset of a HeLa cell imaged at a resolution of 2s per stack on a spinning disk microscope. To download this dataset (~100MB), run:

```
kitDataTest()
```

Track this movie via

```
kitRun
```

using the default options selected in the GUI.

Load the relevant jobset and job via:

```
jobset = kitLoadJobset();
job = kitLoadJob(jobset,1);
```

Diagnostic plots

```
kitDiagnoseAndTest
```

Evaluate each step of the detection and tracking algorithm to show how many kinetochore sisters are detected, tracked, and grouped at each stage. This is helpful to identify drops in performance at a certain step which can indicate how to therefore improve tracking performance. Example: `kitDiagnoseAndTest(job,1,0)` Note that since the original spot detections before the refinement step (eg. refinement of locations with a Gaussian mixture model) are not stored in the jobset object. Therefore for this evaluation the initial detections need to be performed again which can be time consuming. It may be useful to obtain and store these via `[spots, movie, ~] = kitExampleDetectionTest(job);` if repeating this evaluation. The stored spot locations can then be used via `kitDiagnoseAndTest(job,1,0,spots,movie,1)`

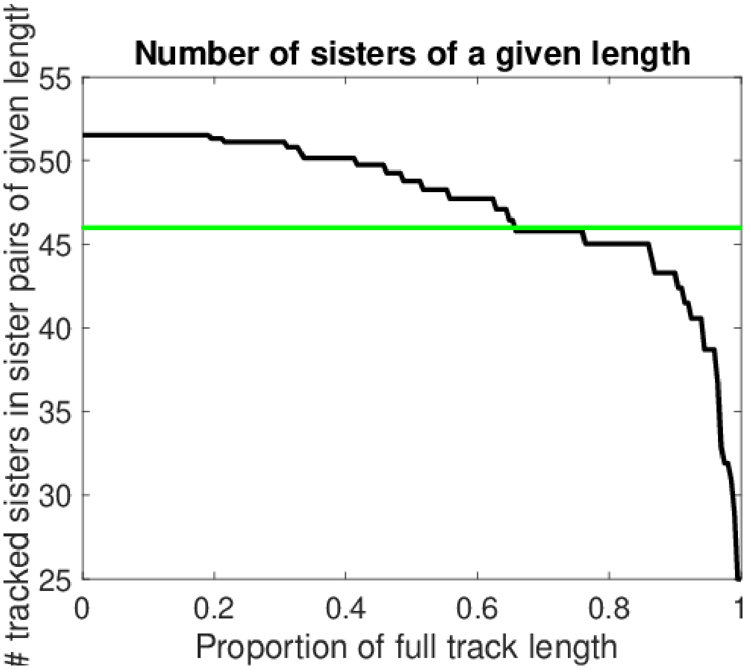

This is a key plot showing performance of each step of the algorithm, where the colour of the crosses corresponds to the step of the algorithm. If few spots (say less than half of the expected number) are found, then the initial detection of spots may be problematic. Check that imaging quality looks reasonable in the raw movies. Try deconvolving the movies. If the whole cell is not in the field of view of the movie, then this could also explain a low number of detected spots, but the user may want to adjust the expected number of spots in the GUI or use the histogram detection method instead or the adaptive method.
If a reasonable number of spots are detected, but few spots are tracked then this suggests an issue with linking spots between frames. This could be due to large movements of kinetochores between frames (insufficient temporal resolution). Try using a smaller time step between movie frames if possible. If kinetochore pairs can be tracked well but not grouped into sister pairs, then check there is a sufficent duration of metaphase frames in the movie. In prometaphase, kinetochore pairs will not be aligned to the metaphase plate. There is an optiion in the GUI to use alignment in pairing sister kinetochores. Turning off this option can help to pair (unaligned) kinetochores in prometaphase. Anaphase frames cannot be used to pair kinetochore sisters. Manual pairing is available for fixed data (ie. without time series).

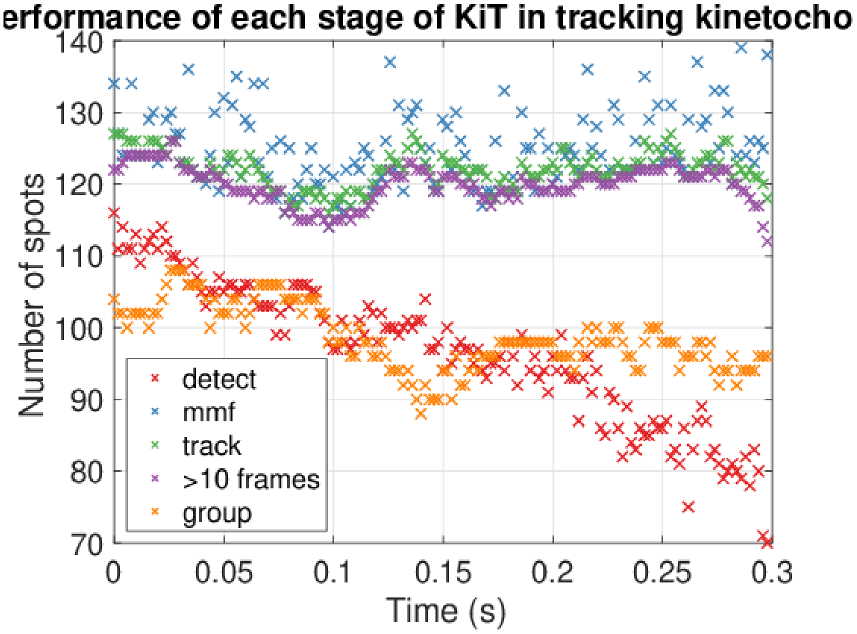

This plot shows the number of sister kinetochore pairs tracked through a given duration of movie, with lines to indicate the expected number of kinetochore pairs in human cells (46).

```
kitPlotHowManyTracksOfGivenLength
```

Use this to visualise a barcode plot of when tracks start and stop throughout a movie. This can highlight whether high quality tracking has been achieved, whether there is a certain part of the movie whether tracking is worse than elsewhere, or whether there is a constant gain and loss of tracks throughout the movie. Example: `kitPlotHowManyTracksOfGivenLength(job)`

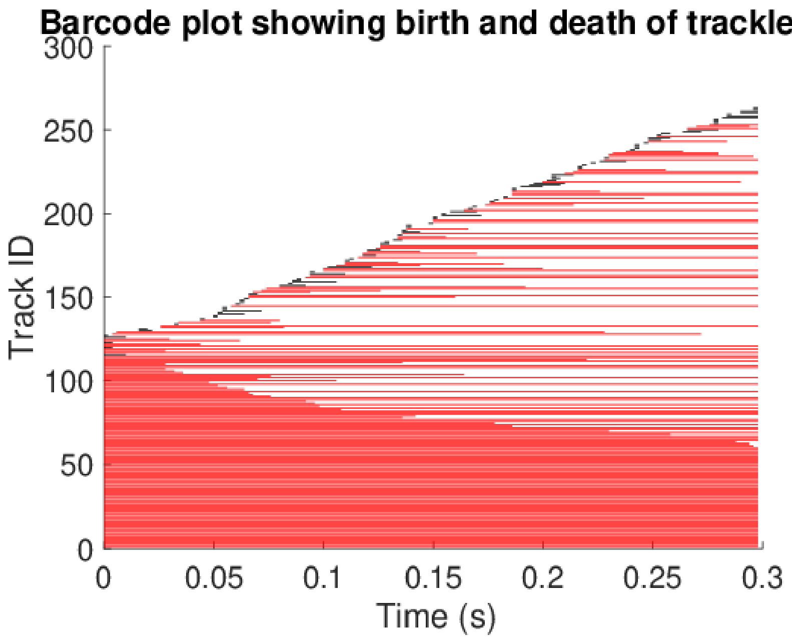

The following plots can be helpful for visualisation as well as diagnosing issues with tracking:

```
kitShowDragonTails
```

Use this to visualise tracks on an image. Helps to verify that a reasonable number of spots have been detected and tracked. Can identify tracks with label switching between kinetochores.

Example: `kitShowDragonTails(job,’usePairs’,0,’crop',0,'timePoint',10,'tailLength',5);` Note that in the test dataset centrioles have also been labelled and have been tracked. We can choose to visualise only paired sister kinetochores: `kitShowDragonTails(job,'usePairs',1,'crop',0,‘timePoint',50,'tailLength',5,'tracks',1:60);`

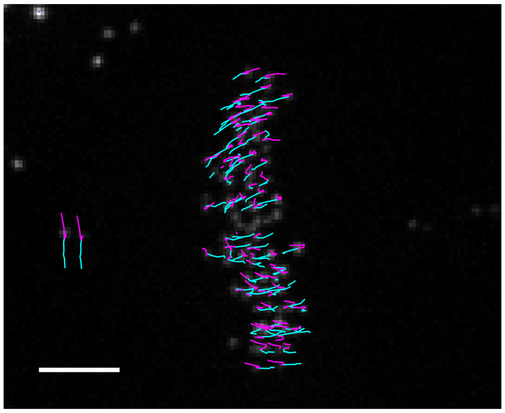

`kitShowSpots`

Use this to visualise detected spots in a frame. A cropped region around each spot is shown. Helpful to check that spots are kinetochores as opposed to background. Biorientated kinetochores are expected to have a Gaussian shaped spot-like signal. Example: `kitShowSpots(job,’timePoint’,10)`

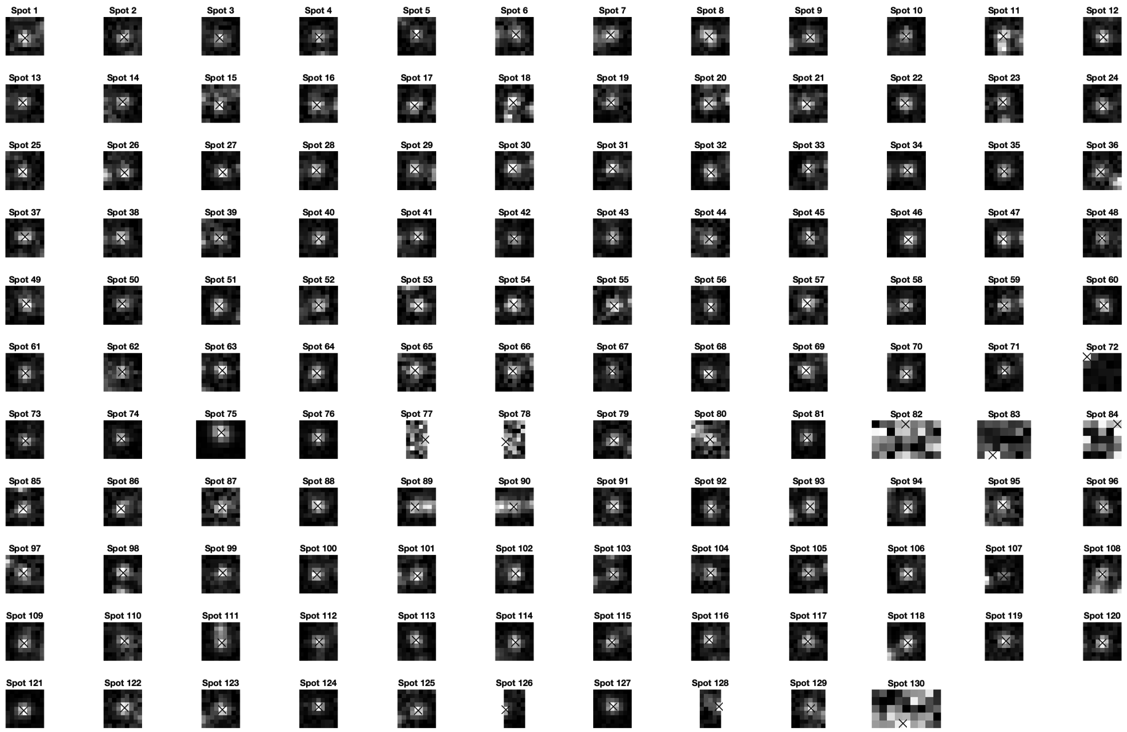


```
kitShowSisters
```

Use this to visualise paired sister kinetochores in a frame. Example: `kitShowSisters(job,'timePoint',10)`

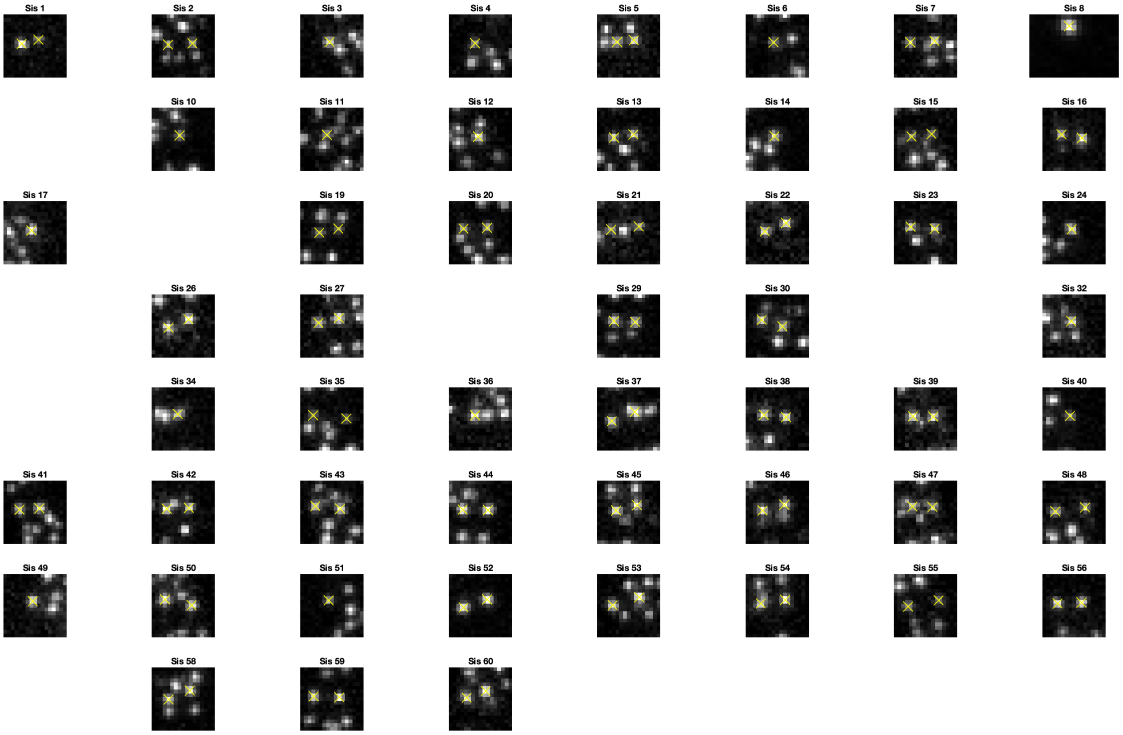
Sis 1
Sis 2
Sis 3
Sis 4
Sis 5
Sis 6
Sis 7
Sis 8
Sis 10
Sis 11
Sis 12
Sis 13
Sis 14
Sis 15
Sis 16
Sis 17
Sis 19
Sis 20
Sis 21
Sis 22
Sis 23
Sis 24
Sis 26
Sis 27
Sis 29
Sis 30
Sis 32
Sis 34
Sis 35
Sis 36
Sis 37
Sis 38
Sis 39
Sis 40
Sis 41
Sis 42
Sis 43
Sis 44
Sis 45
Sis 46
Sis 47
Sis 48
Sis 49
Sis 50
Sis 51
Sis 52
Sis 53
Sis 54
Sis 55
Sis 56
Sis 58
Sis 59
Sis 60
